# Electronic Endoscopy in Endoscopic Mucosal Resection (EMR) of Gastric Cancer

**DOI:** 10.1155/DTE.1.209

**Published:** 1995

**Authors:** Minoru Kawaguchi, Ryouichi Misaka, Michiru Yamada, Shouko Midorikawa, Tetuya Sanji, Satoshi Shinohara, Shigefumi Morita, Yutaka Handa, Hiroyuki Ohno, Yasuhiko Saitou, Hajime Yosida, Masahisa Takase, Toshihiko Saitou

**Affiliations:** The 4th Internal Medicine Tokyo Medical College 6-7-1 Nishishinjuku Shinjuku-ku Tokyo 160 Japan

## Abstract

The role in which electronic endoscopy plays is important in EMR. It is useful in diagnosis and treatment of gastric cancer from a clinical viewpoint. EMR with use of electronic endoscopy allows better coordination between the operator and assistants, and thus improves the results further.


*Diagnostic and Therapeutic Endoscopy*, 1995, Vol. 1, pp. 209–216
Reprints available directly from the publisher
Photocopying permitted by license only

© 1995 Harwood Academic Publishers GmbH
Printed in Singapore

### Electronic Endoscopy in Endoscopic Mucosal Resection (EMR) of Gastric Cancer

MINORU KAWAGUCHI, RYOUICHI MISAKA, MICHIRU YAMADA, SHOUKO MIDORIKAWA, TETUYA SANJI, SATOSHI SHINOHARA, SHIGEFUMI MORITA, YUTAKA HANDA, HIROYUKI OHNO, YASUHIKO SAITOU, HAJIME YOSIDA, MASAHISA TAKASE, and TOSHIHIKO SAITOU

*Fourth Department of Internal Medicine, Tokyo Medical College*

*(Received October 7, 1994; in final form December 9, 1994)*

The role in which electronic endoscopy plays is important in EMR. It is useful in diagnosis and treatment of gastric cancer from a clinical viewpoint. EMR with use of electronic endoscopy allows better coordination between the operator and assistants, and thus improves the results further.

**KEY WORDS:** Electronic endoscopy, endoscopic mucosal resection, early gastric cancer

### INTRODUCTION

Endoscopy for digestive tract diseases began with the rigid gastroscope developed by Kussmaul in 1868. Gastrocameras developed in Japan in 1950 came into wide clinical use. Gastrocameras were equipped with a camera and a lamp at the tip of the scope. As the target was directly photographed using the flash mechanism, resolution was high, but a high level of technique also was necessary because photography was performed "blind" under fluoroscopic monitoring.

In 1958, Hirshowitz developed a fiberscope that drastically improved endoscopic examinations. The fiberscopic examination has become essential for daily clinical diagnosis and treatments.

The first electronic endoscope (videoendoscope) was developed by Welch-Allyn, Co. in the United States in 1983 (1,2). Subsequently, improved instruments were developed by Machida and Toshiba (TV endoscope), Fuji Shashin (electric videoendoscope), and Olympus (videoimage endoscope).

The electronic endoscope has a different structure from the conventional fiberscope. A charged couple device (CCD), a solid state image device, is mounted at the tip. The device converts the object image into electric signals that are reconstructed as images on the monitor screen. The electronic endoscope yields high-resolution images, simplifies image processing, and enables simultaneous observation by many staff (3–7).

This paper concentrates mainly on the last feature. We report the effectiveness of the electronic endoscope in endoscopic mucosal resection (EMR) in cases of early gastric cancer.

### SUBJECTS AND METHOD

### Subjects

Pathologically demonstrated cases of gastric cancer in which endoscopic mucosal resection was considered indicated. Our indications of EMR are (*a*) cancer lesions less than 2 cm in greatest dimension; (*b*) well differentiated tubular adenocarcinoma (intestinal type adenocarcinoma); (*c*) cancer unaccompanied by peptic ulcer; and (*d*) cancer invasion considered to be limited to the mucosa by

Address for correspondence: Minoru Kawaguchi, M.D., The 4th Internal Medicine, Tokyo Medical College, 6-7-1 Nishishinjuku, Shinjuku-ku, Tokyo 160, Japan.

clinical examination. All the above conditions must be satisfied.

If the above conditions are met, EMR can be employed in inoperable cases. In such cases, however, if some of the cancer lesion remains, it is treated by laser vaporization.

Forty-four cases were treated by EMR from January 1988 to December 1992 (including 5 cases of focal cancer in adenoma). The male to female ratio was 8 to 3, and the average age was 69.6 (Table 1).

### Method

First the lesion is marked by a diathermy probe, then a submucosal injection of 20% glucose is made to elevate the lesion. The lesion is then strangulated and resected.

### *Marking the lesion*

The tip of a diathermy probe is used to lightly mark the mucosa surrounding the lesion. Heating with a power of 15 to 20J turns the mucosa white, clearly indicating the site of the lesion and leaving a safety margin of several mm. The entire circumference of the lesion then is marked.

### *Local submucosal injection*

Because physiological saline can diffuse rapidly after injection resulting in loss of the swelling of the lesion, 20% glucose was used. Sometimes adrenaline is added to prevent hemorrhage. The local injection elevates the lesion for easy strangulation with the snare and also completely separates the lesion from the muscularis propria. Caution is needed in selecting the injection target. It is essential to accurately predict the direction of the resulting swelling and to choose an injection point that does not hide the lesion. Excessive injection results in overinflation of the lesion and may make strangulation with the snare difficult.

### *Lifting the lesion*

The FG-7L grasping forceps (Olympus Co.) were used. The lesions frequently are friable, and pulling up of the lesion is difficult when only a part of the cancer is held up to lift up normal mucosa immediately adjacent to the lesion. In addition, it is important to try to estimate what the shape will be after it is lifted. The snare should strangulate the entire marked area.

**Table 1** Materials (1988.1~1992.12)

| = *EMR* = | | | |
|---|---|---|---|
| *Diagnosis* | *No.* | *Age (average)* | *M/F ratio* |
| Carcinoma | 44 | 69.6 | 8:3 |

### *Resection*

Resection was accomplished by passing coagulation current from the UES-10 high frequency coagulation apparatus (Olympus). An Olympus GIF-2T10 endoscope was used before introduction of the electronic endoscope, but the Olympus GIF2T-200 electronic endoscope has been used since 1991.

### RESULTS

### CR Rate

Cases in which absence of cancer was recognized endoscopically and biopsy specimens were negative were classified as CR. Complete resection of cancer by a single EMR procedure was possible in 21 (47.7%) of 44 cases, and complete elimination by additional treatments (reEMR or laser, etc.) was possible in 16 cases. Totally, 37 of 44 cases were efficient, and the efficacy rate was 84.1% (Table 2). Four (90.1%) of 7 cases in which cancer remained were operated on under laparotomy (submucosal invasion in 3 cases and a significant amount of tumor remained in 1 case to the extent that complete resection by additional EMR was regarded as impossible). Additional treatment is now being given to the remaining 3 cases. (Table 3)

### Efficacy Rate by Histological Type (Table 4)

Of 41 well differentiated tubular adenocarcinoma, 20 (48.8%) were resected by a single EMR. In the other 21 cases, the lesions of 14 cases disappeared following additional treatments. The overall efficacy rate was 82.9%. On the contrary, in 3 cases of moderately differentiated tubular adenocarcinoma, the efficacy rate was 100%, including additional treatments.

**Table 2** Efficacy rate

| | | |
|---|---|---|
| * Single resection | 21/44 | (47.7%) |
| * Additional treatment | 16/44 | (36.4%) |
| Total | 37/44 | (84.1%) |

**Table 3** Lesions not resected by single EMR

| | |
|---|---|
| Cancer cured with additional treatment | 16 lesions |
| * Laser irradiation | 11 |
| * re EMR | 3 |
| * Others | 2 |
| Cancer still remained (during treatment) | 3 lesions |
| Surgery | 4 lesions |
| * Cancer invaded to submucosal layer | 3 |
| * Much amount of remained | 1 |

**Table 4** Efficacy rate by histological type and macroscopic type

| | *Elevated Type* | *Depressed Type* | *Combined Type* | |
|---|---|---|---|---|
| Well Differentiated | 22/26 (84.6) | 11/13 (84.6) | 1/2 (50) | 34/41 (82.9) |
| Moderately Differentiated | 1/1 (100) | 2/2 (100) | — | 3/3 (100) |
| Totals | 23/27 (85.2) | 13/15 (86.7) | 1/2 (50) | 37/44 (84.1) |
| | | | | ( )% |

### Efficacy Rate by Macroscopic Type (Table 4)

There were 27 elevated type cases, of which 12 cases were resected by a single EMR and cancer disappeared by additional treatment in 11 other cases. The efficacy rate for this group was 85.2%. There were 15 depressed type cases, of which 9 cases were resected by single EMR and cancer disappeared by additional treatment in 4 other cases. The efficacy rate in this group was 86.7%. There were only 2 cases of combined type, in one of which cancer invaded submucosally and operated. The efficacy rate was 50%.

### Efficacy Rate According to Location (Table 5)

The efficacy rate according to location was studied according to the CMA classification of the Japanese Gastric Cancer Research Society. In the A region, 20 of 22 cases were treated effectively, and efficacy rate was 90.9%. In the M region, effectiveness was seen in 11 of 14 cases, the efficacy rate being 78.6%. In the C region, the efficacy rate was 75% (6/8). As for the anterior wall, 11 of 12 cases were treated effectively, and the efficacy rate was 91.7%, 83.3% (10/12) for the lesser curvature, 63.6% (7/11) for the posterior wall, and 100% (9/9) for the greater curvature. These results demonstrated that EMR was effective for small gastric cancers in the antrum and greater curvature, but that lesions in the posterior wall or in the C region were difficult to treat. In noneffective cases of submucosal invasion, EMR itself was not the problem but the depth of invasion before the procedure was misjudged. The 4 noneffective cases, apart from submucosal invasion cases, were located from angular portion of the posterior wall in the corpus.

### CASE REPORT

### Case 1

A 76-year-old woman with type I early gastric cancer inoperable because of complication by severe liver cirrhosis is presented. The diagnosis made by biopsy before EMR was tubular adenoma with severe atypia.

Figure 1 shows an elevated lesion in the greater curvature of the gastric antrum. A marking was made around this lesion after sprinkling a dye (indigocarmine) (Fig. 2). After marking, 20% glucose was injected into the submucosal layer to separate the lesion from the muscularis propria. The lesion, including the marked area, was strangulated with a snare and excised by coagulation electric current. After ensuring that there was no hemorrhage from the cut site, the excised mucosa was collected using basket forceps. Endoscopy was carried out 1 week later (Fig. 3) to determine the presence or absence of residual lesion. However, it is usually difficult to visually ensure the absence of residual lesion because ulcer occur by this time. This makes it necessary to take biopsies along the entire circumference of the lesion. EMR had to be repeated in this case to remove residual cancer in the oral site of the anterior wall.

Histopathological diagnosis was well differentiated tubular adenocarcinoma and intramucosal carcinoma.

**Table 5** Efficacy rate by location

| | *A* | *M* | *C* | |
|---|---|---|---|---|
| Anterior wall | ○○<br>▽■ | ○○○○○<br>▽ | ○○ | 11/12 (91.7) |
| Lesser curvature | ○○○○○<br>▽▽▼ | ●<br>▽▽ | ○ | 10/12 (83.3) |
| Poterior wall | <br>▽▽ | ○○●● | ●●<br>▽▽□ | 7/11 (63.6) |
| Greater curvature | ○○○○<br>▽▽▽▽ | ○ | | 9/9 (100) |
| | 20/22 (90.9) | 11/14 (78.6) | 6/8 (75) | 37/44 (84.1) |
| | | | | ( ) % |

○ elevated type ,▽ depressed type, □ combined type.

●▼■ ineffective lesions

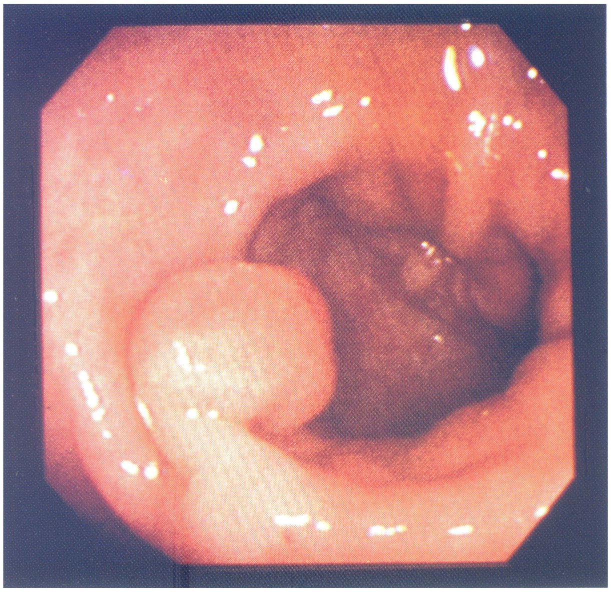

**Figure 1** An elevated lesion was detected in the greater curvature of the gastric antrum.

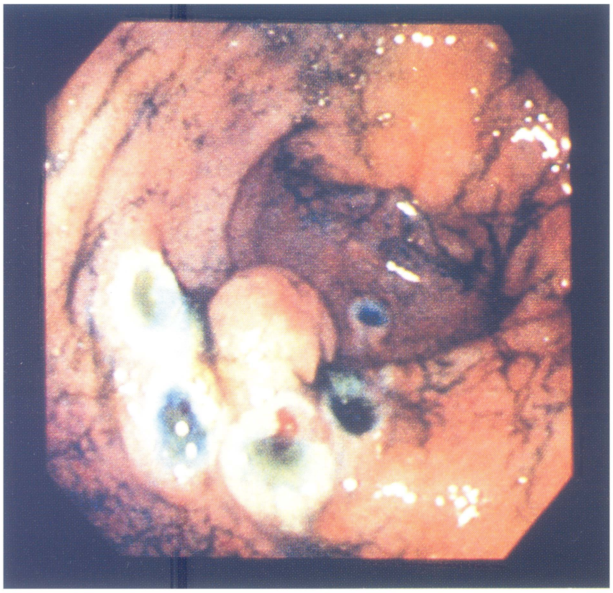

**Figure 2** A marking was made around the lesion after sprinkling a dye.

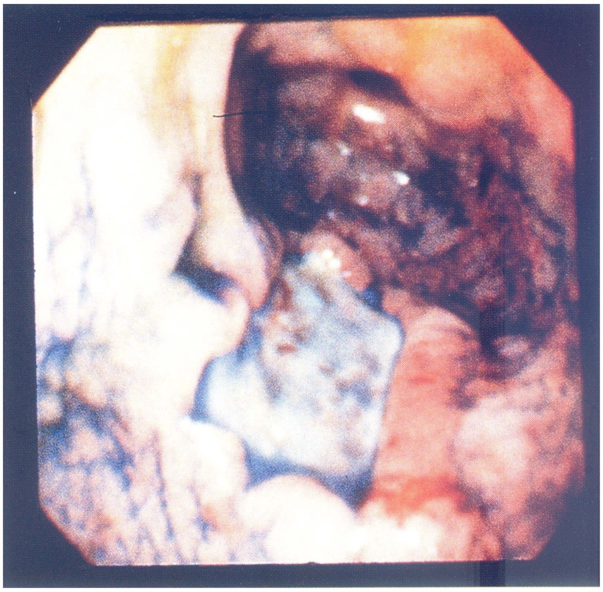

**Figure 3** Endoscopy was carried out 1 week after.

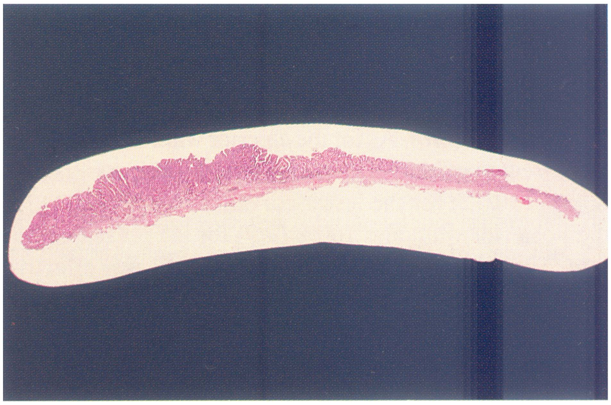

**Figure 4** Histopathological diagnosis was well differentiated tubular adenocarcinoma.

Carcinoma was detected up to the stump in the first material obtained (Fig. 4).

### Case 2

A 72-year-old woman with IIa type early gastric cancer inoperable because of to chronic renal failure and advanced age is presented. An elevated lesion with a wide base and irregular surface was observed in the fornix (Fig. 5). The lesion was clearly demarcated upon a dye (Fig. 6). The diagnosis made based on biopsy before EMR was papillary adenocarcinoma. The circumference of the lesion was marked. After submucosal injection of 20% glucose, the lesion together with an area adjacent to marking was excised with a snare (Fig. 7). Endoscopy was carried out 1 week later. No residual cancer was detected.

In histopathological examinations, a cancer focus was noted (Fig. 8). The diagnosis of papillary adenocarcinoma with a depth of invasion of intramucosa was made.

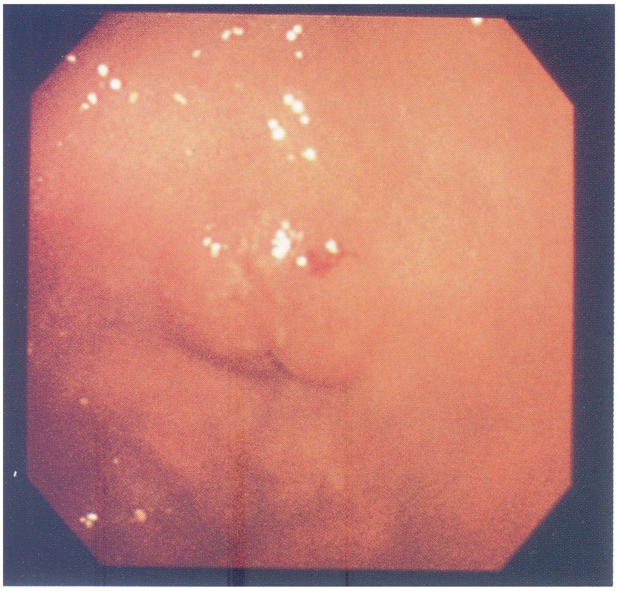

**Figure 5** IIa type early gastric cancer was observed in the fornix.

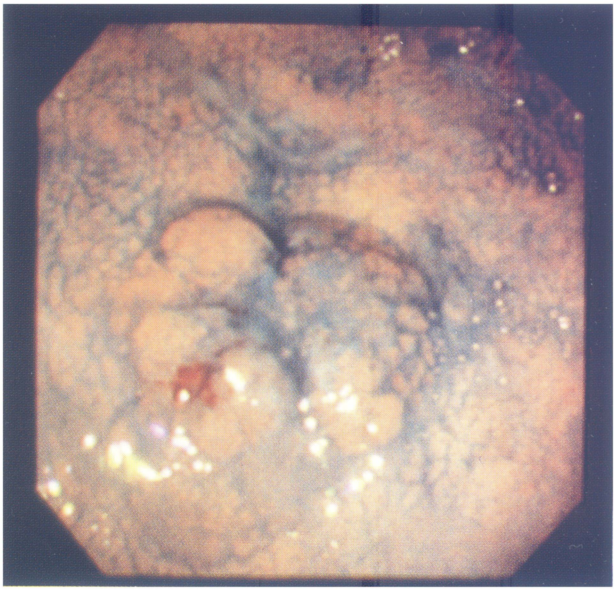

**Figure 6** Endoscopic findings by sprinkling a dye.

### Case 3

EMR was undertaken on request of the patient in 64-year-old man with IIc type early gastric cancer. A shallow, depressed lesion was detected in the anterior wall in the antrum (Fig. 9). The diagnosis of well differentiated tubular adenocarcinoma was made based on biopsy results.

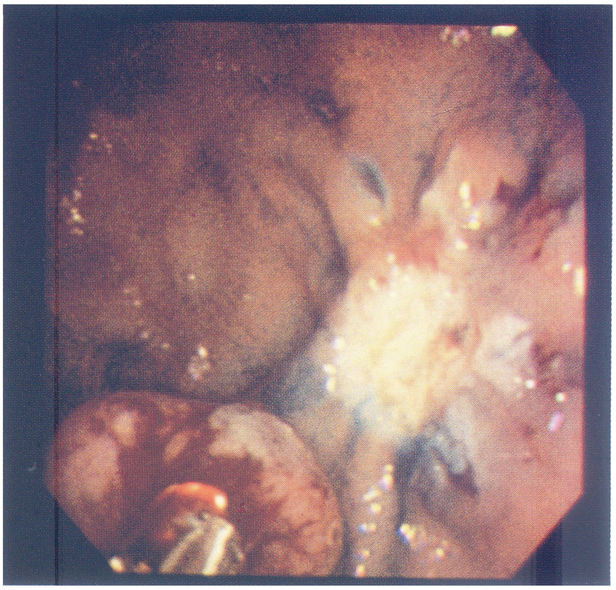

**Figure 7** The lesion was excised with a snare.

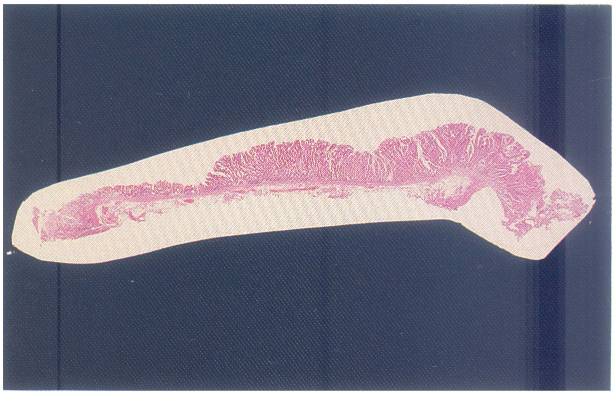

**Figure 8** Histopathological diagnosis was papillary adenocarcinoma.

Endoscopic hemostatic procedure was necessary, because moderate hemorrhage occurred on excision of the lesion (Fig. 10). Endoscopy carried out 1 week later showed a large ulcer, but there no hemorrhage, and no residual cancer was detected.

### Case 4

A 72-year-old man with IIc type early gastric cancer is presented. EMR was performed at the of the request of the patient. An irregular, depressed lesion was detected in the anterior wall in the gastric angle (Fig. 11). The diagnosis, based on biopsy, was moderately differentiated

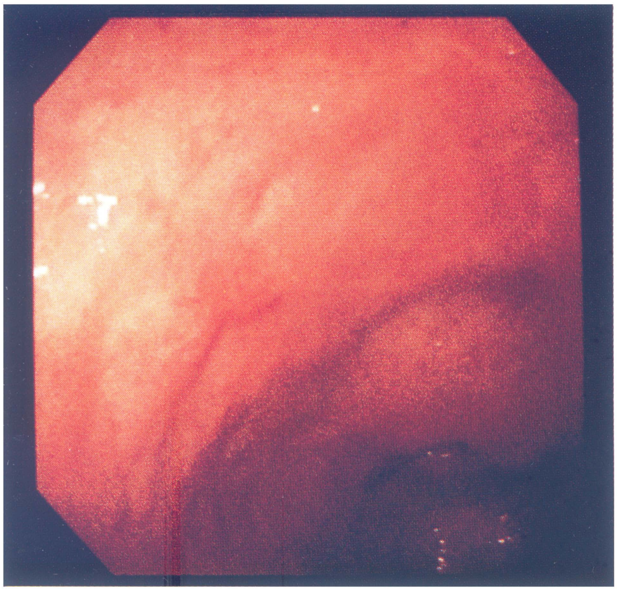

**Figure 9** IIc type early gastric cancer was detected in the anterior wall in the antrum.

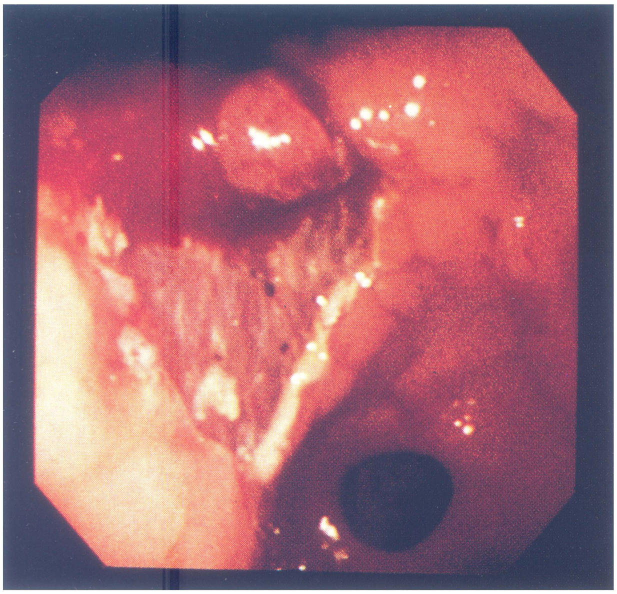

**Figure 10** Hemorrhage occurred on excision of the lesion.

tubular adenocarcinoma. Although there was folds convergence, EMR was considered possible, because the lesion was elevated by submucosal injection of 20% glucose (Fig. 12).

Histopathological examination made after EMR indicated the presence of moderately differentiated tubular adenocarcinoma with a depth of invasion of intramucosa.

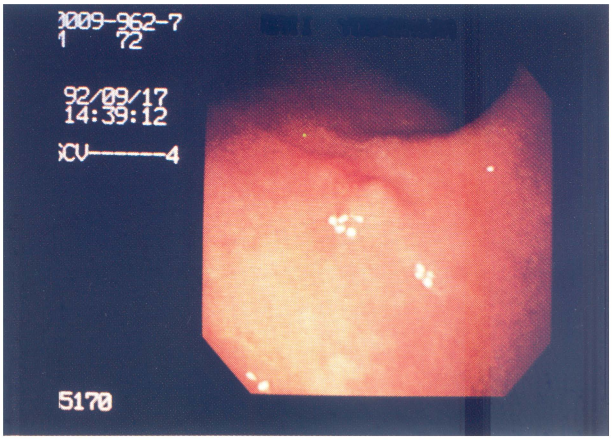

**Figure 11** IIc type early gastric cancer was detected in the anterior wall in the gastric angle.

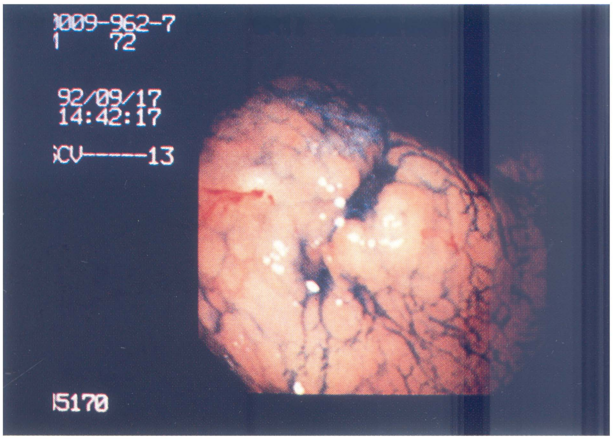

**Figure 12** The lesion was elevated by submucosal injection of 20% glucose.

No evidence of cancer was noted in the stump (Fig. 13). Rupture of the muscularis mucosae and slight fibrosis in the submucosal layer were observed (Fig. 14). However, EMR seemed possible in the presence of such superficial changes.

### DISCUSSION

The electronic endoscope (videoendoscope) has a different structure from conventional fiberscopes (1,2). Theoretical differences between electronic endoscopes and fiberscopes are that (*a*) electronic endoscope has good resolution; (*b*) image processing is possible; (*c*) the monitor image can be observed simultaneously by many staff and students; (*d*) mechanical trouble is less common; and (*e*) it has excellent filing capabilities.

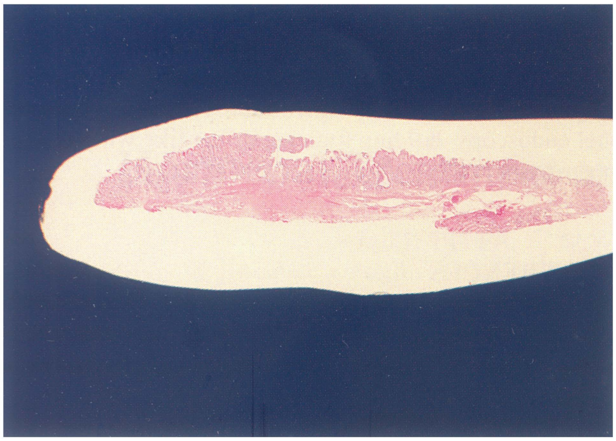

**Figure 13** Rupture of the muscularis mucosae and slight fibrosis in the submucosal layer were observed.

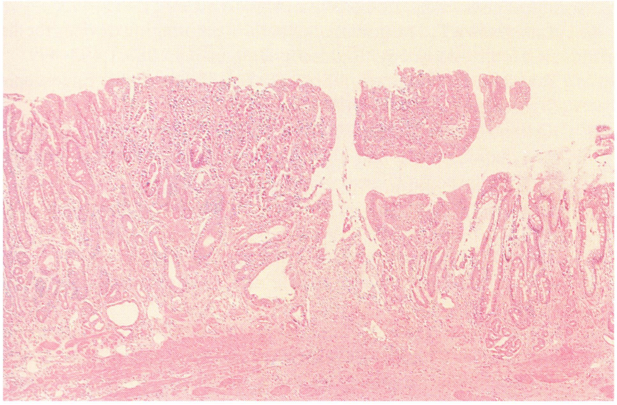

**Figure 14** Histological diagnosis was moderately differentiated tubular adenocarcinoma.

Because the instrument has a large number of pixels, theoretically it should have good resolution (6,7). However, it is the authors' impression that it does not have so high resolution. In addition, very close objects can be too bright and distant ones too dark. Furthermore, the sequential imaging-type electronic endoscope has the disadvantage that picture occurs aberration. Electronic endoscopes do not have better resolution than fiberscopes at present.

Edge emphasis processing and color enhancement processing are examples of image processing made possible by electronic endoscopes. In addition, the infrared can be employed for diagnosis sensitivity of the CCD (8). However, these are not yet applicable for clinical use, because of the need for more image processing equipment and the difficulty in real time image processing.

One great advantage is that the monitor images can be observed by many simultaneously. Although fiberscope images also can be monitored on the screen by connecting the instrument with a television system, the resulting image is poorer than with the electronic videoendoscope image. The mounting of the eyepiece of the fiberscope with the television adapter makes it easy. To emphasize the value of electronic endoscope in treatments of gastric cancer, EMR (endoscopic mucosal resection) was reported in this paper in which cooperation between the operator and the assistants and coworkers is important, omitting image processing topics of the electronic endoscope.

EMR is done using two channel endoscope. Coworking of the operator and assistants is described according to procedure of EMR. Marking is done only by the operator. The assistant regulates the temperature, observing degree of cloudiness of the marking. In local injection, timing between the operator and the assistants is important. The examination goes rapidly when the assistant operates the needle, watching the monitor screen without directions of the operator. The resection is performed by three staff members including the operator who inserts the snare and holds forceps through two forceps inlet of the two-channel endoscope. In addition the assistant to the snare operator must pay close attention when holding up the lesion and resecting it by high-frequency current. The examination proceeds rapidly than before by watching the monitor screen (holding up and resecting was done by oral direction of the operator in fiberscope system).

We have already treated cases of EMR (47 cases of cancer, 38 cases of adenoma, 5 cases of focal cancer in adenoma). We experienced two complications of perforation and hemorrhage. The patient with perforation underwent immediate surgery, and the patient with hemorrhage underwent endoscopic ethanol injection. Fiberscope was used in these two cases, and no complications were experienced after introducing electronic endoscope. This fact demonstrates value of electronic endoscope in EMR.

EMR is presented as strip biopsy by Tada (9). It has since been tried in many hospitals and applied to the lesion in colon and esophagus. We also applied it to lesions in the stomach in 1988, and then to those in the colon and esophagus. The purpose of applying EMR to the gastric cancer is complete resection of the lesion; that is, complete cure. District application is needed for the purpose. Application of EMR was discussed many, times at conferences of the Endoscope Institute. At present, the following four conditions are believed to be applicable to cases of EMR (10): (*a*) well differentiated tubular adenocarcinoma; (*b*) within 2 cm; (*c*) without ulcer; and (*d*) cancer invasion limited to the mucosa.

However, EMR can be used in patients in whom conventional surgery is contraindicated because of age or other reasons, if conditions (*c*) and (*d*) are satisfied. In this case, however, the lesion may remain. The remaining lesion is cured by additional treatments including reEMR.

Our results showed an efficacy rate of 84.1%, which is almost the same as that reported by others (11,12). However, complete resection rate by EMR at the first trial is slightly lower. This may be attributable to wide application of EMR in our hospital and insufficient experience in EMR. Analyzing the results by histological type, the efficacy rate of well differentiated tubular adenocarcinoma is 82.9% and that of moderately differentiated tubular adenocarcinoma is 100%. As the number of the latter is small (only 3 cases), no difference may exist between histological types. However, EMR is not applied to poorly differentiated adenocarcinoma or signet-ring cell carcinoma at all (13).

There was no difference between efficacy rates by macroscopic type (elevated type is 85.2% and depressed type 86.7%). Because there is an absolute condition that the depressed lesion not be accompanied by ulcer, many of them were small and nearly flat. There were only 2 combined types. In one, invasion of a part of cancer into the submucosal layer was observed after resection, and it was treated surgically. Because cancer sometimes invades deeper than visual finding in combined type, very careful examination is needed and the resected specimen should be pathologically investigated. As for efficacy rate by location, results from gastric anglus to the posterior of corpus were bad. It is difficult to observe this portion with front view endoscope even in usual routine examination, and it seems that the disadvantage of front-view endoscope directly influences the results. It is considered that oblique view or side view two-channel endoscopy improves the results. In addition, shortening the hard portion of the tip may improve the results. This is a disadvantage of electronic endoscopy and, development of new CCD is necessary.

Because EMR is done with a two-channel endoscope, we feel that some locations are difficult to treat. However, the result is gradually improved by treating while watching the monitor screen. Further improvement in two-channel endoscopy, holding forceps, or the like will improve the results.

### REFERENCES

1. Sivak MV, Fleischer DF. Colonoscopy with a videoendoscopy: preliminary experience. Gastrointest Endosc 1984;30:1–5.
2. Classen M, Phillip J. Electronic endoscopy of the gastrointestinal tract: initial experience with a new type of endoscope that has no fiberoptic bundle for imaging. Endoscopy 1984;16:16–19.
3. Yao T, Okada M, et al. Current status of electronic endoscope. Stomach Intestine 1987;22:17–25.
4. Kohri Y, Katio T, et al. Electronic endoscopy and image processing of endoscopic pictures. Endoscopia Digestiva 1989;1:437–444.
5. Ikuno Y. Aspects of electronics in the potential ability of videoimage endoscope. Endoscopia Digestiva 1989;1:485–490.
6. Yanagisawa T. The potential of solid state imaging devices for the electronic endoscopy. Endoscopia Digestiva 1989;1:493–499.
7. Takeshita F. The potential of electronic endoscopy. Endoscopia Digestiva 1989;1:501–508.
8. Kawano S, Sato N, et al. Analysis of mucosal and submucosal hemodynamics using and electronic endoscope within an infrared light. Endoscopia Digestiva 1989;1:461–467.
9. Tada M, Shimada M, et al. New technique of gastric biopsy. Stomach Intestine 1984;19:1107–1116.
10. Tada M, Murakami A, et al. Long-term follow-up study of strip biopsy therapy for early gastric cancer. Endoscopia Digestiva 1990;2:1515–1519.
11. Uchizawa M, Mirao M, et al. Evaluation of ERHSE (endoscopic resection with local injection of hypertonic saline epinephrine solution) for the treatment of early gastric carcinoma. Stomach Intestine 1991;26:275–282.
12. Ohizumi H, Matsuda T, et al. Endoscopic resection for early gastric cancer: the actual procedure and clinical evaluation. Stomach Intestine 1991;26:289–300.
13. Nakamura K, Ishidoh T, et al. Partial resection of gastric mucosa: evaluation from pathological point of view. Stomach Intestine 1988;23:411–417.

